# Type I interferon in HIV treatment: from antiviral drug to therapeutic target

**DOI:** 10.2217/hiv.09.8

**Published:** 2009-04-30

**Authors:** Adriano Boasso

**Affiliations:** 1 >Department of Immunology, Division of Investigative Science, Faculty of Medicine, Imperial College, Chelsea & Westminster Hospital, 369 Fulham Road, London SW10 9NH, UK. Tel.: +44 208 746 5993; Fax: +44 208 746 5997; a.boasso@imperial.ac

**Keywords:** anti-IFN-α antibodies (MEDI-545), chloroquine/hydroxychloroquine, CpG oligodeoxynucleotides, HIV/AIDS, IFN-α/β, imiquimod/resiquimod, immunoregulatory sequences, plasmacytoid dendritic cells, soluble CD4-Ig, Toll-like receptors

## Abstract

Type I interferons (IFNs) are soluble molecules that exert potent antiviral activity and are currently used for the treatment of a panel of viral infections. In the case of HIV, the use of type I IFN has had limited success, and has almost been abandoned. During the last decade, a series of studies has highlighted how HIV infection may cause overactivation of type I IFN production, which contributes to the exhaustion of the immune system and to disease progression. This review describes the transition from the proposed use of type I IFN as antiviral drugs in HIV infection, to the idea that blocking their activity or production may provide an immunologic benefit of much greater importance than their antiviral activity.

**Figure 1.**
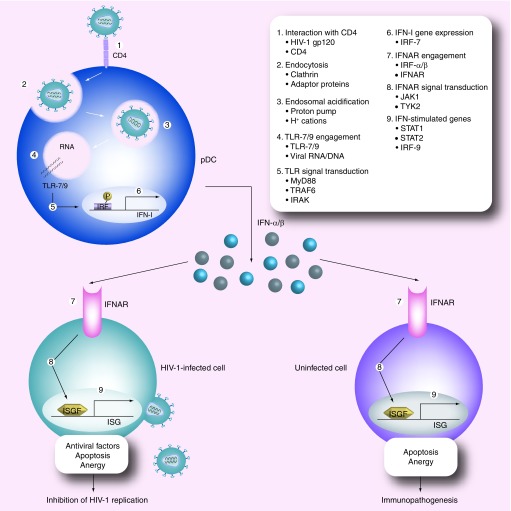
HIV-mediated plasmacytoid dendritic cell activation, and antiviral or pathogenic effects of type I interferon. pDC engulf HIV after its interaction with CD4. HIV is processed in the endosomes and viral RNA is recognized by TLR-7/9. Transcription of type I IFN genes is induced. Secreted type I IFN engage the IFNAR on target cells exerting their antiviral activity on HIV-infected cells, but contributing to apoptosis and anergy of uninfected lymphocytes. Numbers in the figure refer to key steps of plasmacytoid dendritic cell activation and type I IFN signaling pathways, and the key molecules involved are indicated. IFN: Interferon; IFNAR: Type I interferon receptor; IRF: Interferon regulatory factor; ISG: Interferon stimulated gene; ISGF: Interferon stimulated gene factor; pDC: Plasmacytoid dendritic cell; TLR: Toll-like receptor.

The term ‘interferon’ was coined by Alick Isaacs and Jean Lindenmann in 1957 to describe the soluble factor responsible for interfering with the growth of influenza virus *in vitro* [1]. Different types of interferons (IFNs) have been identified and, with the exception of IFN-γ (type II IFN), are referred to as type I IFNs [2]. IFN-α (which comprises at least 22 glycoproteins produced by post-translational modification of 13 gene products), IFN-β, -ε, -κ, -ω and -υ are the six species of type I IFN found in humans. In this review, the term ‘type I IFN’ will be used to describe IFN-α and IFN-β, the best characterized of this family of soluble molecules. The identification of the cellular sources of type I IFN, their pathways of induction and mechanisms of antiviral activity provided a number of potential molecular targets for therapeutic approaches.

The panel of genes expressed by the target cells upon engagement of the receptor for type I IFN (IFNAR) contribute to generate an intracellular environment that antagonizes viral replication [3,4]. 2–5-oligoadenylate synthetase and RNase L inhibit viral replication by degrading viral RNA [5]. The dsRNA-activated kinase PKR contributes to inhibit protein synthesis and activates NF-kB, favoring apoptosis of the target cells [6–10]. In addition, type I IFN play an important role in the development of T-cell-mediated antigen-specific immune responses by stimulating antigen presentation [11–13] and polarizing CD4 T-helper cells toward a Th1 response, which is efficient against viral infection [14,15].

There are two major pathways that result in the induction of type I IFN during viral infections. Retinoic acid-inducible gene-I-like receptors are cytoplasmic sensors that can recognize viral dsRNA in infected cells [16–18]. Toll-like receptor (TLR)-3, -7 and -9 are located in the endosomal compartment of specialized cells and recognize dsDNA, ssRNA and unmethylated CpG-rich DNA, respectively. These nucleic acids are common constituents of the genome of most viruses, which can be engulfed by endocytotic cells [16–18]. In particular, plasmacytoid dendritic cells (pDC) express both TLR-7 and -9 and are specialized in the recognition of viruses and in the production of very large amounts of type I IFN during innate immune responses [18,19]. Myeloid DC express TLR-3 and can also produce type I IFN [18].

Type I IFN responses can also be activated by stimuli that are not directly associated to viral components. For example, host-derived nucleic acids, derived from necrotic or apoptotic cells, can be engulfed by endocytotic cells and induce type I IFN production by TLR-dependent or -independent mechanisms [18,20,21].

## Type I IFN responses to HIV

Similar to other viruses, HIV type 1 is recognized by the molecular sensors that activate innate immune pathways resulting in type I IFN production. The ability of type I IFN to inhibit HIV replication and the possibility to manipulate pDC responses have been a fertile ground for supporting potential therapeutic approaches aimed at taking advantage of this natural antiviral system. However, whilst therapies based on the antiviral activity of type I IFN proved to be efficacious in the treatment of other viral infections, their impact on the course of HIV disease has been minimal. Novel approaches are now being considered in light of the improved understanding of HIV pathogenesis. In particular, consideration is being given to the possibility that pDC and type I IFN may contribute to sustain the status of chronic activation, which eventually results in the dismay of the immune system [22]. Inhibiting pDC activation and type I IFN production or signaling may therefore represent a new avenue for therapeutic approaches.

### ▪ Anti-HIV activity of type I IFN

Since the first reports on the inhibitory effect of type I IFN on HIV replication, the obvious suggestion was made that such molecules could be used as therapeutic agents to control the infection [23]. Obstruction of HIV replication by type I IFN appears to occur at different stages of the viral cycle, with effects which may be specific for certain cell types [24]. Thus, the general antiproliferative and proapoptotic effect of type I IFN are only partially responsible for their anti-HIV activity. Of particular interest is the fact that monocytes/macrophages respond to type I IFN by upregulating and activating the apolipoprotein B mRNA-editing enzyme-catalytic polypeptide-like 3G (APOBEC3G), an intracellular enzyme that exerts potent antiretroviral activity [25]. APOBEC3G blocks HIV replication prior to integration of the proviral DNA into the target cell’s genome [26], but its induction by type I IFN is not observed in CD4 T-cell lines [27]. The cell-specific induction pattern of APOBEC3G may partially explain why type I IFN primarily inhibits the assembly and release of HIV in chronically-infected CD4 T cells [24,28], whilst reducing the relative levels of viral RNA and integrated provirus in macrophages [24,29,30]. Recent evidence also suggest that type I IFN may exert anti-HIV activity through inducing tetherin, a molecule which impedes virus release from the cell surface and is antagonized by the HIV accessory protein Vpu [31,32]. The anti-HIV activity of type I IFN has also been confirmed *in vivo* in the artificial system of SCID mice transplanted with human cell lines or primary leukocytes [33].

### ▪ Type I IFN production during HIV infection

HIV is a strong activator of pDC and potent inducer of type I IFN production *in vitro*, yet studies on type I IFN responses in HIV-infected patients have given apparently contrasting results on whether the type I IFN system is hypo- or hyperfunctional during chronic infection (reviewed in [34]). The interaction of HIV with CD4 expressed on pDC is required to allow its endocytosis and subsequent presentation of the viral nucleic acid to endosmal TLR-7 and -9, essential for pDC activation [35]. The peculiar ability of HIV to interact with CD4, combined with the rapid endocytosis of CD4 by pDC, lead to the suggestion that HIV may provide a particularly strong and protracted trigger for pDC [22]. Recognition of HIV RNA by TLR-7 is probably the main route of HIV-induced pDC activation [35,36], but a role for TLR-9 cannot be excluded.

pDC from HIV-infected patients are refractory to *in vitro* re-stimulation with TLR-7 and -9 ligands or with HIV itself, and the frequency of circulating pDC is reduced in peripheral blood during HIV infection [34]. The dynamics of circulating pDC have been described to follow the changes observed in CD4 count during HIV infection. Thus, circulating pDC decrease during primary HIV infection, and this reduction is partially prevented by early HAART [37]. Efficient HAART treatment also recovers the levels of circulating pDC and their responsiveness to TLR stimulation in chronically HIV-infected patients, but not in patients in whom the CD4 count restoration is impaired [38]. Although these observations suggest an impairment of pDC functionality and type I IFN responses during HIV infection, a number of recent reports outlined how the reduction and apparent hyporesponsiveness of circulating pDC are both consequences of chronic HIV-induced hyperactivation. Plasma levels of type I IFN were described to increase rapidly in macaques upon simian immunodeficiency virus (SIV) infection [39,40]. Abel and colleagues observed that the induction of type I IFN responses occurred early during acute infection and persisted throughout the course of disease [39]. Expression of type I IFN and IFN-inducible genes was observed in different lymphoid tissues, and associated with high tissue SIV RNA rather than control of viral replication [39]. The chronic triggering of pDC may lead to their relocation to lymphoid tissues and to the induction of an apparent refractory status induced through negative feedback [41,42]. These findings reconcile with earlier observations that the levels of type I IFN and type I IFN-regulated genes, including those associated with apoptotic pathways, are increased in peripheral leukocytes and lymphoid tissues from HIV-infected patients [43–45]. Therefore, during HIV infection, pDC may be chronically activated rather than hypofunctional, and production of type I IFN chronically induced rather than inhibited. The hypothesis that chronic pDC activation and type I IFN production may play a pivotal role in driving the immune dysfunction observed during HIV infection has brought alternative therapeutic methods to the attention of scientists and clinicians. The possibility that approaches aimed at blocking type I IFN or their signaling pathways may provide an immunologic benefit during HIV infection is supported by animal models showing that the prolonged stimulation of pDC causes dysfunctions similar to those observed in HIV-infected patients [46,47]. The expression of markers of chronic T-cell activation, considered a hallmark of HIV disease progression, may be at least, in part, dependent on HIV-induced type I IFN [48,49], and pDC from SIV natural hosts, which do not develop immunodeficiency and do not show signs of chronic immune activation, produce far less IFN-α/β in response to TLR-7 and -9 stimulation compared with human or macaque pDC [50]. Furthermore, recent evidence from a nonhuman primate study shows that early accumulation of pDC at the initial site of infection in the vaginal mucosa drives the affluence of CCR5^+^ CD4 T cells, which are targets for SIV infection at this site, ultimately favoring the systemic diffusion of the virus [51].

Activation of the type I IFN pathway by HIV can occur also through alternative pathways, directly involving gp120 signaling to monocytes/macrophages [52–54]. Furthermore, the extensive apoptosis of immune cells and necrotic damage at mucosal and lymphoid tissues during HIV infection may result in the induction of type I IFN by endogenous nucleic acids as described above [18,20,21]. Mobilization of microbial components from the disrupted gut mucosa may also contribute to activate the innate immune system, as suggested by the direct correlation observed between plasma levels of lipopolysaccaride and IFN-α in HIV-infected patients [55]. Although lipopolysaccaride is not likely the direct inducer of type I IFN, its translocation from the intestinal mucosa to the circulation may correlate with the presence of other molecules of viral or bacterial origin capable of inducing IFN-α/β [55].

Thus, the classic idea of type I IFN-based therapy as an antiviral approach against HIV replication, similar to what has been successfully done for other viral infections, is being reconsidered in favor of the exact opposite approach, aimed at inhibiting the overactivated immune mechanism that may have deleterious long-term consequences for the host. It is likely that, *in vivo*, type I IFN have both antiviral and pathogenic consequences at the same time; the key question that still needs to be answered is whether one effect prevails over the other or whether their relative contribution changes in different stages of the disease, and therefore which therapeutic approach is preferable. Preclinical studies and clinical trials following one or the other hypothesis are ongoing. A schematic representation of the pathway of HIV-mediated pDC activation and its potential antiviral and pathogenic consequences is shown in Figure 1. Table 1 shows a list of approved or prospective drugs which act on the type I IFN pathway and are or may be considered for the treatment of HIV infection.

## HIV therapy: type I IFN as antiviral drugs

The well described antiviral activity of type I IFN posed the basis for them to be considered as therapeutic agents against HIV infection. In later years, the more profound understanding of the mechanisms underlying their induction and production have added new possibilities for therapeutic approaches aimed at mimicking or triggering these natural antiviral molecules.

### ▪ Recombinant, pegylated & natural IFN-α

Type I IFN-based therapy relies on recombinant technology to produce recombinant IFN-α2a, IFN-α2b and, more recently, their pegylated forms, which have longer a half-life *in vivo* [56]. Natural IFN-α, purified from appropriately stimulated human leukocytes, is also approved for therapy, and has the advantage of containing all IFN-α subtypes, which also preserve their glycosylated structure [57]. Recombinant IFN-β is also available, but it is regularly used for the treatment of multiple sclerosis rather than for viral infections [58]. Administration of IFN-α, in its recombinant, pegylated or natural form, is probably the most direct attempt to take advantage of the antiviral activity of these molecules. No significant differences have been reported between recombinant and natural IFN-α. Recombinant IFN-β1b has rarely been considered as an antiviral agent [201].

The use of IFN-α to treat HIV-infected patients has been considered and tested since the first half of the 1980s [59]. Most clinical trials have focused on co-infection with HIV and other viral opportunistic pathogens such as Kaposi’s sarcoma virus, HCV and HBV. A beneficial effect of IFN-α was immediately recognized in the treatment of Kaposi’s sarcoma in HIV patients in whom the number of circulating CD4 T cells was at least partially preserved [60]. By the early 1990s several independent studies indicated IFN-α as an efficient anti-tumor drug in HIV patients with Kaposi’s sarcoma who had CD4 counts higher than 200 cells/mm^3^ (reviewed in [61]). The anti-tumor effect was often associated with a reduction in the circulating levels of HIV p24 antigen (determination of plasma viral RNA by PCR was not yet available), suggesting a partial anti-HIV effect [61]. IFN-α has also been successfully used in the treatment of viral hepatitis, associated or not with HIV infection, and pegylated IFN-α is still an integral part of the treatment of choice for HBV and HCV (reviewed in [62,63]). Despite this early interest in type I IFN as a therapeutic approach against HIV infection, the fact that their efficacy was limited to immunocompetent patients together with the introduction of azidothymidine first and then HAART have reduced the enthusiasm and dampened the interest for this approach. Of the registered clinical trials on type I IFN therapy for the purpose of treating primarily HIV rather than other opportunistic infections, only four are still active and only two of these are still actively recruiting [202,203]. A recent study on chronically SIV-infected macaques showed that short-term treatment (daily dose for 14 weeks) with pegylated IFN-α2a had no effect on plasma viremia, despite inducing transient increases in the expression of type I IFN-inducible genes [64]. Yet, recent studies on co-infection with GB virus C in HIV-infected patients receiving HAART have shown that the activation of genes associated with the type I IFN pathway correlated with the better prognosis normally observed in these patients [65,66]. These studies suggest that stimulation of the type I IFN system may be beneficial when HIV replication, the principal chronic stimulus for pDC activation, is efficiently suppressed by HAART. In addition, a recent study involving HCV/HIV-co-infected patients showed that administration of pegylated IFN shortly after HAART interruption delayed the rebound of HIV plasma viral load [67].

### ▪ TLR-7 & TLR-9 agonists

Different steps of the pathway of pDC activation and type I IFN signaling could be considered as a potential target for inducing or enhancing an antiviral response (Figure 1). However, as of today, only agonists of IFNAR, in the form of recombinant or natural IFN-α and IFN-β, are regularly used as therapeutic agents, even when conditions other than HIV infection are considered. The direct stimulation of pDC through TLR agonists, particularly targeting TLR-9 and -7, may provide an alternative to the use of recombinant or natural type I IFN. The potential for the use of TLR-7 and -9 agonists in different clinical indications has been recently reviewed elsewhere [68]. This review will focus specifically on their potential and their limitations as therapeutic agents in HIV infection. CpG oligodeoxynucleotides (ODNs) are potent agonists of TLR-9 and induce type I IFN production both *in vitro* and *in vivo* [69]. The clinical applications for which CpG ODNs are currently being considered involve primarily their use as adjuvants in vaccination strategies, taking advantage of their ability to boost the innate immune response, which in turns favors the development of efficient vaccine-induced adaptive immunity [68]. TLR-7 agonists, such as imiquimod and resiquimod, are commonly used in topically applied cream formulations for the treatment of skin conditions such as virus-induced genital warts [70,71]. However, preparations of both imiquimod and resiquimod for oral administration have been developed and have undergone clinical trials for chronic infections [72].

In the case of HIV infection, CpG ODNs could be considered not only as adjuvants in vaccine-based therapy or prophylaxis, but also as therapeutic agents taking advantage of the antiviral activity of type I IFN. *In vivo*, CpG ODNs were shown to improve vaccine-induced memory T-cell responses, and their application as adjuvants to improve vaccine-induced T-cell responses represents one of their main prospective clinical applications in the setting of HIV infection [73]. Although of interest, these applications inevitably rely on the delivery of an efficient vaccine, which is still not available for HIV. Whether suboptimal vaccine strategies that have already been investigated can be significantly improved by optimizing the adjuvant with which they are paired remains to be determined. A therapeutic approach can be considered based on the potential type I IFN-mediated anti-HIV activity of CpG ODN, as demonstrated for thymic pDC *in vitro* [74]. CpG ODN were also shown to improve the anti-HIV activity of other effector cells of the innate arm of the immune response. For example, the lysis of infected CD4 T cells by natural killer cells is dependent on type I IFN production by pDC, and is therefore positively affected by CpG ODNs *in vitro* [75]. A similar application has been proposed for the TLR-7 agonist imiquimod, which was tested in a Phase I study for administration to asymptomatic HIV-infected patients, but did not result in a consistent reduction in viral load, despite increasing circulating levels of type I IFN in all treated patients [76]. The question can be raised as to which advantages and disadvantages the administration of TLR-7 and -9 agonists may have compared with IFN-α/β. Although the proposed therapeutic ability of molecules that stimulate pDC depends largely on the induction of type I IFN production, this approach may have a broader biological effect than the simple use of recombinant or natural IFN-α. Thus, by stimulating pDC, TLR-7 and -9 agonists may promote not only the production of type I IFN, but also the maturation of pDC into effector cells with cytotoxic, antigen presenting and migratory ability [36]. In particular, the expression of the chemokine receptor CCR7 on activated pDC may contribute to their migration to lymph nodes [77], which are important reservoirs for HIV [78]. Although many of the phenotypic and functional changes induced by TLR-7 and -9 agonists in pDC depend themselves on type I IFN signaling, direct stimulation of pDC via TLR-7 or -9 may still provide an anatomic advantage over the simple administration of IFN-α, in that activated pDC will produce antiviral type I IFN directly in the sites where HIV is actively replicating, whereas the dynamics with which IFN-α distributes in lymphoid tissue is hard to predict. On the other hand, chronic HIV infection results in progressive impairment of pDC responsiveness to TLR-7 and -9 stimulation [41], which may undermine the potential efficacy of treatments which target these receptors. This hyporesponsiveness is in part caused by viral proteins, such as gp120-mediated inhibition of TLR-9 activation [79], and in part by the fact that HIV itself is a powerful and persistent stimulus for pDC, causing them to be refractory to further stimulation due to type I IFN-mediated negative feedback [41]. This last observation constitutes a strong criticism to the use of TLR-7 and -9 agonists or IFN-α/β as antiviral treatments of HIV infection: if HIV itself is a stimulus powerful enough to dampen the responsiveness of pDC to external stimulation, why is this not sufficient to exert antiviral effect? Also, in such an overstimulated environment, is it really beneficial to deliver additional pressure to the immune system? The potential risk of pDC overstimulation are epitomized by studies performed on murine models, in which repeated administration of TLR-7 or -9 agonists resulted in severe impairment of immune function [46,47].

## HIV therapy: type I IFN as theraputic targets

Ideally, the optimal therapy against a viral infection is the one aimed at clearing the organism of the infectious agent or, at least, reducing the replication and spread of the virus. In this respect, the approaches described so far, based on the antiviral activity of type I IFN, are the most obvious way of taking advantage of these molecules. However, HIV may represent an exception compared with other viruses, in that the disease associated with the infection is characterized by a progressive exhaustion of the immune system, as a consequence of its chronic hyperstimulation [80,81]. Furthermore, the antiproliferative and proapoptotic effects of type I IFN on T lymphocytes and other immune cells, support the hypothesis that chronic activation of pDC and of the type I IFN system may be an important contributor to the immunodeficiency caused by HIV [22].

### ▪ Re-evaluation of the role of type I IFN in HIV infection

Although the mechanisms through which HIV causes progressive impairment of immune function are still not fully elucidated, it is well accepted that the lymphotropic nature of HIV and its ability to chronically trigger immune responses eventually results in the exhaustion of the immune system [22,80]. pDC and type I IFN may play a pivotal role in this process, and their activation by HIV may be favored by the binding of gp120 to CD4 [22,35]. Furthermore, HIV differs from most chronic viral infections in that it preferentially replicates in lymphoid tissues and produces cell-free virions throughout the course of infection, therefore providing a constant and systemic stimulus for pDC (reviewed in [82]). Chronic overproduction of type I IFN has been suggested to contribute to:

- ▪ Selective ligand/receptor-mediated CD4 T-cell apoptosis (reviewed in [83]);
- ▪ Suppression of T-cell responses via inhibitory ligands [49];
- ▪ Induction of T-cell activation markers [48];
- ▪ Generalized lymphadenopathy, similar to what is observed in murine models [46,47].

Furthermore, a recent study showed that natural hosts of SIV, such as sooty mangabeys, are characterized by reduced type I IFN production by pDC in response to TLR-7 and -9 engagement compared with rhesus macaques [50]. This difference correlates with the absence of AIDS in SIV natural hosts [50], suggesting that in disease-susceptible hosts the chronic activation of pDC and subsequent type I IFN production may play a major role in the exhaustion of the immune system [22]. Concomitantly, the role of type I IFN in raising and sustaining both T-cell and B-cell responses [84–87] may directly favor the chronic hyperactivation of adaptive immune responses, repeatedly reported as a main contributor to HIV pathogenesis [81,88–90]. Moreover, a topical anti-inflammatory agent, which prevented pDC accumulation in the vaginal mucosa, protected rhesus macaques from SIV infection through the vaginal route [51], underscoring the important role that pDC have in also favoring the recruitment of activated T cells at the site of infection, thus fueling the initial burst of viral replication [51].

Taken together, these observations posed the rationale for changing the therapeutic approach to HIV infection with regards to the type I IFN system. Thus, different interventions are currently under investigation with the goal of counteracting or suppressing the deleterious consequences of HIV-induced pDC activation on the immune system [22].

### ▪ Blockers of IFN-α/IFNAR interaction

The biologic effects of type I IFN are mediated through the engagement of their specific receptor on target cells. *In vitro*, blocking antibodies against IFN-α or IFNAR prevent most of the negative effects of HIV-induced pDC activation, including T-lymphocyte apoptosis [44], immunosuppression and expression of activation markers [48,49]. In mouse models, the deleterious consequences of repeated administration of TLR-7 and -9 are limited or absent in IFNAR knockout mice [46,47], confirming the pivotal role of IFNAR engagement by type I IFN in the pathogenic effect of chronic pDC stimulation. Hence, the possibility of utilizing blocking antibodies against type I IFN or their receptor IFNAR is a relatively simple and straightforward therapeutic approach.

Antibodies which act as antagonists of IFNAR have been widely used *in vitro* [36,48,49], but their development into therapeutic agents has not yet reached the phase of human clinical trials. On the contrary, fully humanized monoclonal antibodies against IFN-α (MEDI-545, Medimmune, Gaithersburg, MD, USA) are under clinical experimentation for use in the treatment of autoimmune conditions such as systemic lupus erythematosus (SLE). Two Phase I trials have been completed (SLE and chronic plaque psoriasis [204,205]); two Phase I trials (SLE and dermatomyositis or polymyositis [206,207]) and one Phase II trial (SLE [208]) are currently recruiting. The result of trials on anti-IFN-α antibodies to SLE may be particularly informative with regard to their potential application in HIV infection. Thus, SLE and HIV disease share not only the over activation of the pDC/type I IFN system [22,91], but also some of their most defining immune abnormalities (reviewed in [92]), such as:

- ▪ Decreased CD4 T-cell count
- ▪ Expression of T-cell activation markers
- ▪ Impaired T-cell responses to antigens and mitogens
- ▪ B-cell dysfunction
- ▪ Imbalance of type 1 and type 2 cytokines
- ▪ Increased risk of opportunistic infections

If an improvement of these conditions can be induced in SLE patients by anti-IFN-α antibodies treatment, the possibility of a positive impact on HIV infection using the same or similar approaches may be concrete.

One of the disadvantages that blockade of type I IFN or IFNAR may have is the lack of specificity of the approach, in that ablation of type I IFN signaling, if achieved, may negatively affect the balance of the immune response by disturbing the physiologic immunoregulatory function of type I IFN besides relieving their pathogenic pressure. Also, the ablation of type I IFN responses may impair the efficient immune response to other infections, for which a functional IFN-α/β system is a vital requirement.

A different approach aimed at counteracting the overproduction of IFN-α was attempted in the mid–late 1990s with encouraging results [93,94]. HIV-infected patients were immunized against IFN-α2b in order to induce the production of neutralizing antibodies which would prevent type I IFN signaling. The immunization was efficacious in the Phase I/II studies, and immunized patients showed better preservation of CD4 count and delayed clinical deterioration [93]. In the Phase II/III study that followed, a suboptimal immunization protocol (three oil-adjuvanted injections were used in the Phase II/III trial, compared with six or seven in the Phase I/II study) was used, and production of neutralizing antibodies was induced only in one-third of the enrolled patients [94]. However, patients who responded to immunization with an increase in anti-IFN-α antibodies showed a lower rate of disease progression [94]. Owing to the introduction of protease inhibitors in HIV therapy during the period of the trial, more than 60% of the patients enrolled were elected to add these drugs to their regimen, and experienced the marked improvements in clinical progression normally observed with HAART [94]. This change in therapy made it impossible to achieve the expected end points of the study, prompting to halt further follow-up studies [94]. Nevertheless, these studies suggest that interventions aimed at interfering with type I IFN signaling may prove beneficial in the treatment of HIV disease.

Alternative approaches to interfere with the type I IFN system may target the mechanism through which HIV activates pDC. The characterization of pDC as major producers of type I IFN and the dissection of their activation pathway have highlighted a number of potential molecular targets for inhibiting HIV-mediated pDC activation. Prospective therapeutic agents directed against the pDC activation pathway are presented below.

### ▪ TLR-7 & TLR-9 antagonists

pDC sense HIV through intracellular TLR-7 and -9, which have evolved to recognize viral nucleic acids [35]. Nonstimulatory DNA sequences have been identified that can antagonize the stimulatory effect of both TLR-7 and -9 ligands [95,96]. In particular, immunoregulatory sequences (Dynavax Technologies, Berkley, CA, USA) have been shown to block pDC activation by both DNA and RNA viruses, including HIV [50,95]. Similar to anti-IFN-α antibodies, these TLR-7 and -9 antagonists are now being considered for the treatment of SLE [68], and upcoming clinical trials may provide important information on their applicability to HIV infection. However, immunoregulatory sequences also share the same risks highlighted for anti-IFN-α antibodies, in that their prolonged administration, which may be required for chronic conditions such as HIV, may have unpredictable effects on the regulatory balance of the immune system and potentially favor other opportunistic viral infections by impeding innate immune responses.

### ▪ Chloroquine & hydroxychloroquine

The increasing interest in molecules that may interfere with HIV-associated chronic immune activation has attracted the attention of scientists and clinicians towards chloroquine and hydroxychloroquine, two compounds which have been largely used for the treatment of malaria, and that affect the acidification of endosomes in phagocytic cells [97]. Chloroquine and hydroxychloroquine diffuse through the cell membrane and raise the pH of endocytotic vescicles [97]. Besides being necessary for the life cycle of *Plasmodium*(hence the antimalaric activity of chloroquine and hydroxychloroquine), the acidic pH is also required for processing engulfed pathogen and presenting their antigens [98], as well as exposing nucleic acids to TLR-7 and -9 in pDC [99]. The activation of pDC by HIV, and its consequences on the phenotype and function of T cells, are also sensitive to inhibition by chloroquine [35,36,48–50]. Because it is already approved for use in the treatment of malaria and autoimmune disorders such as SLE and rheumatoid arthritis, chloroquine is probably the drug with the most immediate potential for application to HIV treatment. A Phase II/III trial [209] is already recruiting for testing the antiviral and anti-inflammatory effect of chloroquine in HIV-infected patients. Chloroquine presents the additional advantage of having demonstrated an inhibitory effect on HIV replication *in vitro* [100]. Thus, chloroquine may have a beneficial effect on HIV disease by two separate routes: an effect on HIV replication with consequent reduction of the chronic immune-activating viral stimulus, and a direct effect on pDC activation, reducing their responsiveness to HIV and therefore limiting chronic immune activation. Besides the same downsides noted above for other immunoregulatory agents such as TLR-7 and -9 antagonists and anti-IFN-α antibodies, the prospect of long-term administration of chloroquine or hydroxychloroquine must take into consideration the well known adverse effects reported for these drugs, some of which are potentially serious such as severe damage to vision, which can occur with chronic use [101].

Both chloroquine and TLR antagonists have the disadvantage that they would be inefficient in preventing type I IFN production resulting from triggering of retinoic acid-inducible gene-I-like receptors in infected cells [18], gp120 interaction with monocytes/macrophages [52–54] and endogenous nucleic acids resulting from apoptotic or necrotic events [18,20]. The latter mechanism, in particular, has been hypothesized to play a role in the pathogenesis of certain autoimmune disorders, and may also contribute to HIV pathogenesis [18,20].

### ▪ Soluble CD4-Ig & anti-gp120

Production of type I IFN by pDC is part of the normal early innate immune response against viral infections [17,102]. However, during HIV infection, pDC stimulation continues throughout the course of the disease [22,39]. Furthermore, HIV possesses a characteristic feature that distinguishes it from most viruses, in that it engages the target cells via CD4. Blockade of gp120–CD4 interactions inhibits the induction of type I IFN and other pDC-mediated immunoregulatory mechanisms *in vitro* [35,36,103,104]. This interaction can be antagonized in at least two different ways: by using antibodies directed against the HIV envelope protein gp120; or by using a recombinant soluble form of human CD4 (sCD4-Ig) [104]. Both these molecules were tested in the early 1990s for their potential as anti-HIV drugs (Table 1), with particular interest toward sCD4-Ig [105]. However, the limited effect on viral replication (measured as p24 antigen, as HIV RNA measurement by PCR was not yet available) and the almost complete lack of effect on CD4 count [105], rapidly dissipated the enthusiasm over the therapeutic potential of these molecules. Furthermore, the availability of more efficient antiretroviral drugs during the period in which sCD4-Ig was tested, shifted the interest of scientists and clinicians toward more efficient approaches. Nevertheless, extensive studies on the potential of sCD4-Ig as a modulator of the immunologic effects of HIV, rather than viral replication, have never been performed. Thus, such an approach may not have a strong impact on the plasma virus levels, but may prevent or counteract the chronic activation of type I IFN, with important positive consequences on other markers of disease progression, such as T-cell activation and immune function.

The use of specific blockers of HIV–pDC interaction also presents the advantage of not interfering, as far as it can be predicted, with the physiologic function of pDC and type I IFN. Thus, despite being very efficient in inhibiting pDC activation by HIV, there is no reason to believe that sCD4-Ig may affect the ability of pDC to recognize and produce type I IFN in response to other viruses, leaving the capacity of these cells to initiate an early innate immune response against other opportunistic infections intact. In light of these observations, an approach aimed at specifically blocking the initial interaction of HIV with pDC, using sCD4-Ig or other similar molecules, may be the best therapeutic option to interfere with the type I IFN system. Furthermore, directly inhibiting HIV-mediated pDC activation may affect other pathogenic mechanisms that are not strictly dependent on type I IFN signaling, but require pDC activity. Thus, the expression of the immunosuppressive enzyme indoleamine 2,3-dioxygenase is induced in pDC upon exposure to HIV [48,103], and activation of this mechanism may in turn drive the differentiation of regulatory T cells [106], potentially contributing to the generalized impairment of T-cell responses [107,108].

## Conclusions & future perspective

Type I IFN have long been known as potent natural antiviral agents, and therefore considered as potential therapeutic agents to counteract the progression and spread of different viral infections, including HIV. However, the advances made in understanding HIV pathogenesis have forced a re-evaluation of the role that type I IFN may have in this disease, underscoring how their chronic production may have deleterious effects on the immune system. This alternative view nourished the interest for therapeutic agents that inhibit type I IFN production or signaling, with the goal of limiting the immunologic damage that they may cause during HIV infection. It is likely that a balance exists between the beneficial antiviral effect and the deleterious consequences of type I IFN, and that the same balance extends to all prospective treatments that affect the pDC/type I IFN system.

New molecules that modulate pDC responses or type I IFN function are being considered alongside long-known drugs, such as chloroquine, which affect the same pathway. Although blocking the initial interaction of HIV with pDC, for example using sCD4-Ig, may be the best possible approach, the drug which currently appears to be closest to being used for the purpose of inhibiting HIV-induced pDC activation is likely chloroquine. Encouraging results from the ongoing trials in which chloroquine is being tested for HIV infection may open the door not only for testing compounds which are already available, but also for seeking the development of novel therapeutic agents which target the same pathway, for example by inhibiting key molecular components of the TLR-7 and -9 or IFNAR signal transduction pathways.

**Table 1.** Potential therapeutic agents targeting the type I IFN pathway.

**Active compound**	**Target**	**Mechanism of action**	**Clinical application**
***Agonists***			

CpG ODN	TLR-9	Type I IFN induction (therapeutic agent or vaccine adjuvant)	Colorectal and esophageal cancer^*^ Pneumococcus (HIV-associated)^*^ Mycosis Fungoides^*^ Non-Hodgkin Lymphoma^*^ HBV, HCV^*^

Imiquimod and resiquimod	TLR-7	Type I IFN induction	Skin cancers Genital warts Other skin conditions

Natural IFN-α; Recombinant and pegylated IFN-α2a/2b	IFNAR	IFNAR engagement and signaling	HCV, HBV, KSHV Malignancies (several)

Recombinant IFN-β1b	IFNAR	IFNAR engagement and signaling	Multiple sclerosis Cardiomyopathies^*^ KSHV, CMV (HIV-associated)^*^

***Antagonists***			

Soluble CD4 Ig^‡^	CD4	HIV–CD4 interaction blockade	

Anti-gp120^‡^	Gp120	HIV–CD4 interaction blockade	

Chloroquine	Endosome	Prevention of endosomal acidification	Malaria Rheumatoid arthritis Systemic lupus erythematosus Glioblastoma^*^ Metabolic Syndrome X^*^ Chikungunya fever^*^

Anti-IFN-α mAb	IFN-α	IFNAR–IFN-α interaction blockade	Systemic lupus erythematosus^*^ Psoriasis^*^ Dermatomyositis or Polymyositis^*^

^*^Clinical trials ongoing for these conditions. *^‡^Tested for inhibition of HIV replication, not as immunomodulators.* *CMV: Cytomegalovirus; IFN: Interferon; IFNAR: Type I interferon receptor; KSHV: Kaposi’s sarcoma-associated herpesvirus; mAb: Monoclonal antibody;* *ODN: Oligodeoxynucleotides; TLR: Toll-like receptor.*

**Executive summary**

***Type I interferon responses to HIV***

- ▪ Type I interferons (IFNs) exert powerful anti-HIV activity.
- ▪ HIV is a strong inducer of type I IFN production by plasmacytoid dendritic cells (pDC).
- ▪ pDC are overactivated in HIV-infected patients, which may contribute to the chronic activation and exhaustion of the immune system via type I IFN.

***HIV therapy: type I IFN as antiviral drugs***

- ▪ Recombinant, pegylated or natural IFN-α are used as antivirals against hepatitis and Kaposi’s sarcoma viruses, even when associated with HIV infection, but have been shown to have only limited effect on HIV disease itself.
- ▪ Toll-like receptor (TLR)-9 agonists are under trial as adjuvants and TLR-7 agonists are used in the treatment of virus-induced genital warts; both can be considered for HIV therapy as inducers of antiviral type I IFN.

***HIV therapy: type I IFN as therapeutic targets***

- ▪ Recent evidence suggests that overactivation of pDC and type I IFN production may contribute significantly to HIV pathogenesis and disease progression.
- ▪ Antibodies against IFN-α are under clinical trials for the treatment of systemic lupus erythematosus, and may be considered for blocking IFN-α signaling in HIV-infected patients to limit the damage caused by chronic type I IFN production.
- ▪ Antagonists of TLR-7 and -9 have been used *in vitro* to inhibit HIV-induced pDC activation and type I IFN production, but clinical trials have not yet started.
- ▪ Chloroquine and hydroxychloroquine are approved for the treatment of malaria and systemic lupus erythematosus, and may be used to inhibit HIV-induced pDC activation by blocking endosomal acidification. Clinical trials are ongoing.
- ▪ Soluble CD4-Ig was tested in the 1990s as a blocker of HIV infection, but may be reconsidered for its ability to prevent HIV-induced pDC activation.

***Conclusion***

- ▪ The view of scientists and clinicians on the role of type I IFN in HIV pathogenesis has changed: despite their potent antiviral activity, type I IFNs are now considered important contributors to HIV disease progression.
- ▪ The possibility of using recombinant type I IFN as anti-HIV drugs has been replaced by the idea of blocking type I IFN signaling or production to limit the damage they may cause to the immune system.
